# Treatments Outcomes in Histological Variants and Non-Urothelial Bladder Cancer: Results of a Multicenter Retrospective Study

**DOI:** 10.3389/fonc.2021.671969

**Published:** 2021-05-20

**Authors:** Nicolas Epaillard, Pauline Parent, Yohann Loriot, Pernelle Lavaud, E-B. Vera-Cea, Nieves Martinez-Chanza, Alejo Rodriguez-Vida, Clement Dumont, Rebeca Lozano, Casilda Llácer, Raffaele Ratta, Stephane Oudard, Constance Thibault, Edouard Auclin

**Affiliations:** ^1^ Medical Oncology Department, Hôpital Européen Georges Pompidou, AP-HP, Université de Paris, Paris, France; ^2^ Gustave Roussy, Université Paris-Sud, Université Paris-Saclay, Villejuif, France; ^3^ Medical Oncology Department, Hospital del Mar, IMIM Research Institute, Barcelona, Spain; ^4^ Medical Oncology Departments, Jules Bordet Institute, Université Libre de Bruxelles, Brussels, Belgium; ^5^ Medical Oncology Department, Hôpital Saint Louis, AP-HP, Université de Paris, Paris, France; ^6^ Prostate Cancer Clinical Research Unit, Spanish National Cancer Research Centre, Madrid, Spain; ^7^ Genitourinary Oncology Translational Research Group, Instituto de Investigación Biomédica de Málaga (IBIMA), Málaga, Spain; ^8^ Medical Oncology Department, Hopital Foch, Suresnes, France

**Keywords:** urinary bladder neoplasms, variant histology, drug therapy, immunotherapy, mortality

## Abstract

**Introduction:**

Less than one-third of bladder cancers are non-pure urothelial carcinoma [with variant histological (VH) or non-urothelial carcinoma (non-UC)] for which no treatment guidelines are available. We aim to evaluate the efficacy of systemic treatments in VH or non-UC bladder cancers.

**Materials:**

Multicenter retrospective analysis of patients treated for advanced or metastatic VH or non-UC bladder cancers. Primary endpoint was overall response rate (ORR) according to treatment line, regimen and histology subtype. Secondary endpoints were progression-free survival (PFS) and overall survival (OS).

**Results:**

Between 2005 and 2020, 46 patients from seven centers were included. The median age was 66 years (58.75; 74.75), 65.2% were male and 67.2% presented VH. At first line, the ORR for the entire population was 54.4% and median OS was 21.6 months (95% confidence interval [CI]: 14.2-38.6). The ORR of the 37 patients treated with chemotherapy at first line was 62.2% with median PFS and OS of 7.3 (95% CI: 4.5-8.6) and 21.6 months (95% CI: 14.2-35.7), respectively. Dose dense MVAC and platinum doublet chemotherapy had the highest ORR (71.4% and 65.2%). The 9 patients treated with immunotherapy at first line had an ORR of 22.2%, a median PFS of 3.3 months (95% CI:2.3-NR) and the median OS was not reached (95% CI:13.8-NR). Response to treatment varied depending on the histological sub-types and on the treatment type.

**Conclusion:**

Chemotherapy and immunotherapy have shown to be effective in VH or non-UC cancers, a rare histological subtype for which we currently have very little data in the literature.

## Introduction

Bladder cancer represents the sixth most common cancer in Europe with an incidence of 11.3 per 100.000 persons (1). The most frequent histology is urothelial carcinoma (UC) that counts around 60-90% patients (2–6). In the majority of cases, UC is found in its pure form but in around 20% of patients, variant histological (VH) features are observed, such as squamous cell differentiation, glandular differentiation, micropapillary, or nested. The histological variants is the major component in half of these patients (7). Pure non-urothelial carcinomas (non-UC) represent 10% of bladder cancers in western countries, including squamous cell carcinoma, adenocarcinoma and neuroendocrine tumors (8, 9).

Because of their rarity, patients with non-UC or predominant VH are frequently excluded from prospective clinical trials evaluating new drugs. At the localized stage, the prognosis of VH and non-UC bladder cancer is uncertain due to conflicting results from different studies (10–13). For this reason, there are currently no guidelines for the management of pure non-urothelial bladder cancer at this stage. Furthermore, very few data are available for metastatic disease; the literature includes only small retrospective series or case reports (2, 14–17). Due to this lack of data, the management of VH and non-UC is currently extrapolated from UC care (18, 19). We therefore aim to evaluate the efficacy of systemic treatments in VH or non-UC bladder cancer.

## Methods

We retrospectively collected data from all patients treated for advanced or metastatic bladder or upper urinary tract cancer with a VH or non-UC, in seven European hospitals between March 2005 and April 2020 using local databases. Patients with VH were included in the analysis only if they had a variant histopathologic growth pattern as the major component (i.e. > 50%). Patients were excluded if they did not receive systemic treatment for advanced or metastatic disease. Patients were considered as advanced disease if they had positive lymph nodes, absence of distant metastasis and had been treated as in the metastatic setting.

For each patient, data was collected from their medical records and included: age, gender, date of diagnostic, ECOG performance status, primitive tumor location, stage, histology, location of metastasis, presence of surgery or not, hemoglobin, and treatment regimen. We also collected for each treatment line: time to relapse, location of relapse, number of metastasis and tumor response.

The primary endpoint was tumor response (overall response rate - ORR, and disease control rate - DCR) according to treatment line, regimen and histology subtype. Secondary endpoints included progression-free survival (PFS) and overall survival (OS) evaluated by each investigator.

*Statistical analysis:* median (interquartile-range) values and proportions (percentage) were provided for the description of continuous and categorical variables, respectively. Median and proportions were compared using Wilcoxon-Mann-Whitney test and chi2-test (or Fisher’s exact test, if appropriate), respectively.

PFS was defined as time between the first-line treatment initiation and progression, or death, whichever occurred first. Alive patients without progression were censored at the date of their last follow-up. OS was defined as time between the first-line treatment initiation and death from any cause. Patients known to be alive were censored at the date of their last follow-up.

Tumor responses were classified according to the Response Evaluation Criteria in Solid Tumors criteria 1.1. ORR was defined as the sum of complete and partial responses.

All analyses were made with RStudio software. *p*-value ≤0.05 was considered as statistically significant.

## Results

### Demographic and Tumor Characteristics

Between 2005 and 2020, a total of 46 patients were included in the study. Patients’ characteristics are shown in **Table 1**. Most of them were men (65.2%) and the median age at diagnosis was 66 years [IQR: 58.75;74.75]. Bladder was the primitive cancer location for 89.1% (n=41) and upper tract for 10.9% (n=5). Thirty-seven (80.5%) patients were classified as metastatic disease and 9 (19.5%) as advanced disease. The most frequent pathological diagnosis was VH (67.2%, n=31). Non-UC included neuroendocrine carcinoma (24.0%, n=11), adenocarcinoma (4.4%, n=2), squamous cell carcinoma and micropapillary (2.2%, n=1 each).

**Table 1:** Baseline characteristics of the study population.

	Overall	Chemotherapy	Immunotherapy	p
Number	46	37	9	
Sex				1
Male	30 (65.2%)	24 (64.9%)	6 (66.7%)	
Female	16 (34.8%)	13 (35.1%)	3 (33.3%)	
Age at L1, median (IQR)	66 [58.75;74.75]	66 [56;75]	63 [62;69]	0.59
Age				0.72
<65y	22 (47.8%)	17 (45.9%)	5 (55.6%)	
>65y	24 (52.2%)	20 (54.1%)	4 (44.4%)	
Primitive loc.				1
Bladder	41 (89.1%)	33 (89.2%)	8 (88.9%)	
Upper tract	5 (10.9%)	4 (10.8%)	1 (11.1%)	
Histology				0.57
Non-urothelial carcinomas	15 (32.8%)	15 (40.5%)	0	
Adenocarcinoma	2 (4.4%)	2 (5.4%)	0	
Squamous cell carcinoma	1 (2.2%)	1 (2.7%)	0	
Neuro endocrine carcinoma	11 (24.0%)	11 (29.7%)	0	
Micropapillary	1 (2.2%)	1 (2.7%)	0	
Variant histological	31 (67.2%)	22 (59.5%)	9 (100%)	
Prior surgery				0.26
Yes	26 (56.5%)	19 (51.4%)	7 (77.8%)	
No	20 (43.4%)	18 (48.6%)	2 (22.2%)	
At first line-treatment				
Number of metastasic sites				0.57
≤2	25 (86.2%)	18 (90.0%)	7 (77.8%)	
>2	4 (13.8%)	2 (10.0%)	2 (22.2%)	
Metastatic sites	1 [1;2] (0;3)	1 [1;2] (0;3)	1 [1;2] (0;3)	
Visceral				
Yes	15 (32.6%)	12 (32.4%)	3 (33.3%)	
Bones				
Yes	6 (13.0%)	3 (8.2%)	3 (33.3%)	
Exclusive node				
Yes	9 (19.5%)	4 (10.8%)	5 (55.6%)	
Missing	17 (37.0)	17 (45.9%)	0	
Treatment				
**Chemotherapy**	37 (80.4%)	37 (100%)	0	
Platine based (alone or in combination)	32 (70.0%)	32 (86.5%)		
Cisplatin	16 (34.8%)	16 (43.24%)		
MVAC	7 (15.2%)	7 (18.9%)		
Cisplatin Gemcitabine	8 (17.4%)	8 (21.6%)		
VIP	1 (2.2%)	1 (2.7%)		
Carboplatin	15 (32.6%)	15 (40.5%)		
Carboplatin Gemcitabine	9 (19.6%)	9 (24.3%)		
Carboplatin etoposide	6 (13.0%)	6 (16.2%)		
Oxaliplatin Gemcitabine	1 (2.2%)	1 (2.7%)		
Paclitaxel	1 (2.2%)	1 (2.7%)		
Other chemotherapy	4 (8.8%)	4 (10.8%)		
**Immunotherapy**	9 (19.6%)	0	9 (100%)	
ECOG Performance status				0.63
0-1	16 (69.5%)	12 (75.0%)	4 (57.4%)	
≥2*	7 (30.5%)	4 (25.0%)	3 (42.8%)	
Hemoglobin, median (IQR)	11.6 [10.62;13.1]	11.4 [10.4;12.9	13.1 [11;14]	0.31

L1, first-line treatment; y, years; loc, location; MVAC, Methotrexate Vinblastine doxorubicin Cisplatin; VIP, Vinblastine Ifosfamide Cisplatin.

Visceral metastasis site: liver, brain, kidney, lung.

*1 patient in the group chemotherapy was PS 4.

### First-Line Therapy

#### Overall

After a median follow-up of 37 months (95%CI: 22.8-NR), the median OS was 21.6 months (95%CI: 14.2-38.6) (**Table 2**). At first line, the ORR of the global cohort was 54.4% with 2 complete responses (4.4%) (**Table 2**).

**Table 2:** Response and survival endpoints according to the treatment and number of lines in the study population.

	Overall	Chemotherapy	Immunotherapy	p
Number	46	37	9	
At first line-treatment				
Confirmed objective response rate	25 (54.4%)	23 (62.2%)	2 (22.2%)	
Disease control rate	27 (58.7%)	24 (64.9%)	3 (33.3%)	
Confirmed best overall response				0.025
Complete response	2 (4.4%)	1 (2.7%)	1 (11.1%)	
Partial response	23 (50%)	22 (59.5%)	1 (11.1%)	
Stable disease	2 (4.4%)	1 (2.7%)	1 (11.1%)	
Progressive disease	19 (41.2%)	13 (35.1%)	6 (66.7%)	
Overall survival (months)	21.6 (14.2-38.6)	21.6 (14.2-35.7)	NR (13.8-NR)	0.5
Progression free survival (months)	5.6 (3.4-8.5)	7.3 (4.5-8.6)	3.3 (2.3-NR)	0.7
At second-line treatment				
Confirmed objective response rate	11 (37.9%)	10 (35.7%)	1 (100%)	
Disease control rate	19 (65.5%)	18 (64.3%)	1 (100%)	

NR, not reached.

#### Chemotherapy

**Figure 1:**
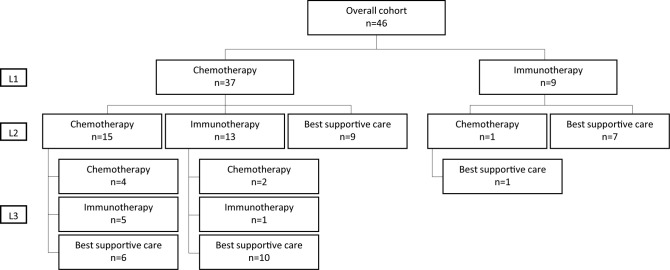
Flow chart. L1, First-line therapy; L2, Second-line therapy; L3, Third-line therapy.

As described in **Table 1** and **Figure 1**, 37 patients were treated with chemotherapy at first line. Most of them were VH (n=22, 59.5%). Several regimens were used, mainly platinum-based chemotherapy (86.5%). With first-line chemotherapy, the ORR was 62.2% with 1 complete response (2.7%) and 22 partial responses (59.5%). Median PFS and OS were 7.3 months (95%CI: 4.5-8.6) and 21.6 months (95%CI: 14.2-35.7), respectively (**Table 2**). As shown in **Table 3**, dose dense (dd) MVAC and platinum doublet seemed to have a higher ORR compared to the other regimens used (ORR: 71.4% and 65.2%, respectively).

**Table 3:** Tumor response according to treatment.

	Number	CR	PR	SD	PD	0RR	Missing
Platinum doublet chemotherapy	23	1 (4.3%)	14 (60.9%)	0	8 (34.8)	15 (65.2%)	0
ddMVAC	7	0	5 (71.4%)	0	2 (28.6%)	5 (71.4%)	0
Other platinum doublet	2	0	0	1 (50%)	0	0	1 (50%)
Other	5	0	2 (40%)	0	3 (60%)	2 (40%)	0
Immune checkpoint inhibitors	9	1 (11%)	1 (11%)	1 (11%)	6 (67%)	2 (22%)	0

Platinum doublet chemotherapy: Cisplatin Gemcitabine, Carboplatin Gemcitabine, Carboplatin etoposide.

ddMVAC, dose dense Methotrexate Vinblastine doxorubicin Cisplatin.

Other platinum doublet: Oxaliplatin Gemcitabine, Vinblastine Ifosfamide Cisplatin

Other: Taxol.

Immune checkpoint inhibitors: pembrolizumab, durvalumab, atezolizumab.

#### Immune Checkpoint Inhibitors

Nine patients (VH 100%) received immune checkpoint inhibitors as first-line treatment (**Table 1** and **Figure 1**): pembrolizumab (77.8%), durvalumab (11.1%) and atezolizumab (11.1%). The ORR was 22.2% with one complete response with pembrolizumab and one partial response with durvalumab (11.1% each). The median PFS was 3.3 months (95%CI: 2.3-NR) and the median OS was not reached (95%CI: 13.8-NR) (**Table 2**).

### Second-Line Therapy

#### Overall

29 patients received a second-line therapy with an ORR of 37.9% (**Figure 1**). The median PFS and OS were respectively 6.0 months (95%CI: 2.8-20.5) and 15.8 months (95%CI: 10.8-NR).

#### Detailed Second-Line Treatments

Among the 37 patients treated with first-line chemotherapy, 28 received second-line treatment, whereas the remaining 9 patients received only best supportive care (**Figure 1**). The second-line ORR and CR rate of those 28 patients were 35.7 and 10.7%, respectively (**Table 2**). Fifteen of the 28 patients received a second-line chemotherapy: platinum based (alone or in combination) (n=7, 25%), paclitaxel (n=6, 21.4%), or other chemotherapy (n=2, 7.1%) with an ORR and CR rate of 26.7% and 0%, respectively. Only 1 patient treated with an immune checkpoint inhibitor at first-line received a second-line treatment, which was chemotherapy (paclitaxel), achieving a complete response.

Among the 37 patients treated with first-line chemotherapy, 13 received an immune checkpoint inhibitor as second-line: pembrolizumab (n=6, 21.4%), nivolumab (n=3, 10.7%), durvalumab (n=2, 7.1%) or atezolizumab (n=2, 7.1%), with an ORR of 46.2% (CR 23.1%).

### Third-Line Therapy

The median PFS and OS at third-line therapy were respectively 2.0 months (95%CI: 1.2-NR) and 12.6 months (95%CI: 4.9-NR). Among the 15 patients treated with chemotherapy at first and second-line, 9 patients received a third-line treatment: 4 received a chemotherapy (paclitaxel 50%, platinum based alone or in combination, 25% each) and 5 were treated with an immune checkpoint inhibitor (atezolizumab or durvalumab, 40% each and nivolumab 20%) (**Figure 1**).

Among the 13 patients treated with chemotherapy at first-line and immune checkpoint inhibitor at second-line, 3 patients received a third-line treatment: 2 received chemotherapy (paclitaxel or carboplatin plus paclitaxel) and one pembrolizumab (**Figure 1**).

### Tumor Response According to Histology and Treatment

**Table 4** details tumor response rates according to histology and treatment type. The VH group had an ORR of 71.4% (no complete response) with MVAC and 61.5% (CR 7.7%) with platinum doublet chemotherapy. Of the 7 VH patients treated with pembrolizumab, 1 (14.3%) reached a complete response (no PR). Among the patients with a histological variant, only 1 had a variant histology exhibiting a neuroendocrine phenotype. This one was treated at first-line with chemotherapy (Cisplatin Gemcitabine) and presented a tumor progression after 5 months. The second line of treatment was a taxane allowing a stability of the disease. His overall survival was 14 months. Regarding non-UC, patients with neuroendocrine carcinoma had an ORR of 57.1% (no CR) with platinum doublet chemotherapy. One patient from each histology (adenocarcinoma, squamous cell carcinoma and micropapillary carcinoma) achieved a partial response.

**Table 4:** Tumor response according to histology and treatment.

Histology	Treatment	Regimen	CR	PR	SD	PD	ORR	Missing
Variant histology 31 (67.4%)	Chemotherapy 22	Platinum doublet chemotherapy 13	1 (7.7%)	7 (53.8%)	0	5 (38.5%)	8 (61.5%)	0
		MVAC 7	0	5 (71.4%)	0	2 (28.6%)	5 (71.4%)	0
		Other 2	0	1 (50%)	0	1 (50%)	1 (50%)	0
	ICI 9	Pembrolizumab 7	1 (14.3%)	0	1 (14.3%)	5 (71.4%)	1 (14.3%)	0
		Durvalumab 1	0	1 (100%)	0	0	1 (100%)	0
		Atezolizumab 1	0	0	0	1 (100%)	0	0
Neuro endocrine carcinoma 11 (24.0%)	Chemotherapy 11	Platinum doublet chemotherapy 7	0	4 (57.1%)	0	3 (42.9%)	4 (57.1%)	0
		Other platinum doublet 1	0	0	1 (100%)	0	0	0
		Other 3	0	1 (33.3%)	0	2 (66.7%)	1 (33.3%)	0
Adenocarcinoma 2 (4.4%)	Chemotherapy 2	Platinum doublet chemotherapy 1	0	1 (100%)	0	0	1 (100%)	0
		Other platinum doublet 1	0	0	0	0	0	1
Squamous cell carcinoma 1 (2.2%)	Chemotherapy 1	Platinum doublet chemotherapy 1	0	1 (100%)	0	0	1 (100%)	0
Micropapillary 1 (2.2%)	Chemotherapy 1	Platinum doublet chemotherapy 1	0	1 (100%)	0	0	1 (100%)	0

Platinum doublet chemotherapy: Cisplatin Gemcitabine, Carboplatin Gemcitabine, Carboplatin etoposide.

MVAC, Methotrexate Vinblastine doxorubicin Cisplatin.

Other platinum doublet: Oxaliplatin Gemcitabine, Vinblastine Ifosfamide Cisplatin.

Other: Taxol.

ICI, immune checkpoint inhibitors.

## Discussion

Our study evaluated the efficacy of first-, second- and third-line therapies in VH and non-UC bladder cancer. For the total VH or non-UC population, the ORR was 54.4% at first-line, with a median OS of 21.6 months. The ORR of first-line chemotherapy was 62.2% with a median PFS and OS of 7.3 and 21.6 months, respectively. Conversely, first-line immune checkpoint inhibitor was associated with an ORR of 22.2%, and a median PFS and OS of 3.3 months and not reached, respectively.

If we compare our results with previously published studies on VH or non-UC bladder cancer (including urothelial and non-urothelial variants), we note that chemotherapy produces tumor responses in the majority of cases, but with variations depending on the histological subtype. Indeed, the reported ORRs are high for small cell carcinoma (75% to 90%) (12, 20), adenocarcinomas (35 to 60%) (2, 10, 21), plasmocytoid (50%) (22), and squamous cell carcinoma (25 to 40%) (2, 15). Reported median OS varies greatly depending on the treatments used and the histological subtype ranging from less than 6 to more than 25 months (2, 10, 12, 15, 17, 20, 21).

To date, few trials have studied immune checkpoint inhibitors in advanced or metastatic VH or non-UC bladder cancer. However, our results seem to be in agreement with previous published data. Sternberg et al. reported an ORR of 9% (CR 1%) with atezolizumab in the SAUL trial (23) whereas Mc Gregor et al. noted an ORR of 37% (CR 5%) with the combination of nivolumab and ipilimumab (24). In both studies, median PFS was less than 4 months. In the neoadjuvant setting, the PURE-01 trial showed a complete pathologic response rate of 16% for patients with predominant VH treated with pembrolizumab (25, 26).

On the other hand, our study shows a similar treatment efficacy compared to what have been published in prospective clinical trials assessing chemotherapy in standard urothelial carcinomas. Indeed, regarding MVAC chemotherapy, the two main prospective trials reported an ORR of around 46 and 58%, with complete response rate ranging from 11 to 23%. Median OS was 15 and 9 months, respectively (27–29). Dose dense MVAC showed the best results, with an ORR 72% and CR rate of 25% (29). Cisplatin plus gemcitabine combination has been evaluated in three prospective clinical trials. ORR ranged from 49 to 66%, CR rate around 20% with a median PFS and OS close to those found with MVAC (8 and 13 months) (28, 30, 31). Carboplatin-gemcitabine combination showed an ORR of 56% including a CR rate of 3%, with a median OS and PFS of 10 and 7 months, respectively (30). Regarding cisplatin-ineligible patients, carboplatin-gemcitabine combination had an ORR of 41% and a low median OS and PFS of 9 and 6 months, respectively (32).

Five trials have studied the use of immune checkpoint inhibitor as first-line treatment for standard urothelial carcinomas: monotherapy (atezolizumab, pembrolizumab, durvalumab) or combination (durvalumab-tremelimumab). These studies demonstrated an ORR from 20 to 30%, a CR rate of less than 10%, and a median PFS and OS of 3 and 16 months, respectively (33–37). These data are very similar to the results of our study: ORR 22.2%, CR rate 11.1%, a median OS NR and a median PFS of 3.3 months. Three clinical trials have studied the role of immunotherapy in patients with previously treated metastatic urothelial carcinoma (38–40). The nivolumab 1 mg/kg plus ipilimumab 3 mg/kg combination seemed to give the most interesting results with an ORR of almost 40% and a median OS of 15 months. These efficacy data are better than in the first-line setting, although no direct comparison can be made between the different studies.

Despite the low number of patients included in our study, we observed a better disease control rate with immunotherapy when administered as second-line (after chemotherapy) than in the first-line setting (ORR = 46.2% versus 22.2%). This sensitizing effect of chemotherapy has already been described in the literature (41).

The main interest of our study lies in the fact that this is one of the first studies describing the treatment efficacy in advanced or metastatic VH or non-UC bladder cancer and detailing the different protocols (chemotherapy, immunotherapy and regimens used) as well as the responses according to the histological subtypes. Indeed, there are only few series for which such precise data are available to date. The strength of our study is its relatively large number of patients included in the context of a rare pathology. The small sample size of the different other published series studying this subject illustrates the difficulty of including patients, due to the rarity of non-UC and VH . However, several limitations can be pointed out. Firstly, this study was a retrospective analysis. This resulted in a number of selection biases or loss of data. In particular, we were not able to gather enough information about the side effects of treatments. Furthermore, the retrospective nature of the study did not allow us to have a pathological central review or to collect molecular data (such as PD-L1, TMB, molecular classification). In addition, although the overall population of our study is large, it is an heterogeneous population from a histological point of view, since we included and analyzed all urothelial subtypes together, thus resulting in small subtypes. However, we have tried to describe the different responses to treatment according to each of the considered subtypes.

## Conclusion

In this multicenter retrospective study, we showed that chemotherapy is an effective treatment option in histological variant and non-urothelial bladder carcinomas. Moreover, despite the small number of patients treated with immunotherapy, the efficacy results were encouraging.

## Data Availability Statement

The original contributions presented in the study are included in the article/supplementary material. Further inquiries can be directed to the corresponding author.

## Ethics Statement

Ethical review and approval was not required for the study on human participants in accordance with the local legislation and institutional requirements. Written informed consent for participation was not required for this study in accordance with the national legislation and the institutional requirements.

## Author Contributions

NE: Writing - original draft, Writing - review & editing. PP: Writing - review & editing. YL: Writing - review & editing. PL: Writing - review & editing. E-BV-C: Writing - review & editing. NM-C: Writing - review & editing. AR-V: Writing - review & editing. CD: Writing - review & editing. RL: Writing - review & editing. CL: Writing - review & editing. RR: Writing - review & editing. SO: Methodology, Writing - review & editing. CT: Methodology, Writing - review & editing. EA: Conceptualization, Formal analysis, Methodology, Writing - review & editing. All authors contributed to the article and approved the submitted version.

## Funding

This research did not receive any specific grant from funding agencies in the public, commercial, or not-for-profit sectors.

## Conflict of Interest

YL reports Grant, personal fees and nonfinancial support from Janssen and MSD; personal fees and nonfinancial support from Astellas, Roche, AstraZeneca, BMS and Seattle Genetics; grant and personal fees from Sanofi; personal fees from Clovis, Incyte and Pfizer. PL reports conflict of interest with IPSEN Mundi Pharma JANSSEN Astellas Pfizer Astra Zeneca. AR-V reports serving in an advisory role for MSD, Pfizer, BMS, Astellas, Janssen, Bayer, Clovis and Roche; receiving honoraria or travel expenses from Pfizer, MSD, Astellas, BMS, Janssen, Astra Zeneca, Roche, Bayer, and Sanofi Aventis; and receiving research funding from Takeda, Pfizer, and MSD. NM-C reports support for research travel from Pfizer, Janssen and Ipsen, and consulting fees for BMS, Pfizer, Sanofi and Bayer. CD reports consulting or Advisory Role: Pfizer. Travel, Accommodations, Expenses: Ipsen, Pfizer, MSD. CL reports Speakers’ bureau: Roche. Travel, Accommodations, Expenses: Astellas Pharma, Angelini Pharma. RR reports Consulting/Advisory board: Pfizer, MSD; Travel, Accommodations, Expenses: Pfizer. SO declares honoraria from Sanofi, Astellas, Janssen, Bayer, Pfizer, Novartis, Ipsen, MSD, BMS, and Astra Zeneca. CT declares Board: BMS, Pfizer, Pfizer, Ipsen, MSD, Astellas, Janssen, AstraZeneca, Merck, Sanofi. Travel: Pfizer, Sanofi, AstraZeneca. Funding: AstraZeneca, Sanofi. EA reports Travel expenses: Mundipharma. Lectures and educational activities: Sanofi Genzymes.

The remaining authors declare that the research was conducted in the absence of any commercial or financial relationships that could be construed as a potential conflict of interest.
